# A Novel One-Transistor Dynamic Random-Access Memory (1T DRAM) Featuring Partially Inserted Wide-Bandgap Double Barriers for High-Temperature Applications

**DOI:** 10.3390/mi9110581

**Published:** 2018-11-07

**Authors:** Myeongsun Kim, Jongmin Ha, Ikhyeon Kwon, Jae-Hee Han, Seongjae Cho, Il Hwan Cho

**Affiliations:** 1 Department of Electronic Engineering, Myongji University, Yongin-si, Gyeonggi-do 17058, Korea; kimms0700@gmail.com (M.K.); hjm2703@naver.com (J.H.); 2 Department of IT Convergence Engineering, Gachon University, Seongnam-si, Gyeonggi-do 13120, Korea; kih3596@gmail.com; 3 Department of Energy IT, Gachon University, Seongnam-si, Gyeonggi-do 13120, Korea; jhhan388@gachon.ac.kr

**Keywords:** harsh environment, space application, 1T DRAM, wide-bandgap semiconductor, high-temperature operation, TCAD

## Abstract

These days, the demand on electronic systems operating at high temperature is increasing owing to bursting interest in applications adaptable to harsh environments on earth, as well as in the unpaved spaces in the universe. However, research on memory technologies suitable to high-temperature conditions have been seldom reported yet. In this work, a novel one-transistor dynamic random-access memory (1T DRAM) featuring the device channel with partially inserted wide-bandgap semiconductor material toward the high-temperature application is proposed and designed, and its device performances are investigated with an emphasis at 500 K. The possibilities of the program operation by impact ionization and the erase operation via drift conduction by a properly high drain voltage have been verified through a series of technology computer-aided design (TCAD) device simulations at 500 K. Analyses of the energy-band structures in the hold state reveals that the electrons stored in the channel can be effectively confined and retained by the surrounding thin wide-bandgap semiconductor barriers. Additionally, for more realistic and practical claims, transient characteristics of the proposed volatile memory device have been closely investigated quantifying the programming window and retention time. Although there is an inevitable degradation in state-1/state-0 current ratio compared with the case of room-temperature operation, the high-temperature operation capabilities of the proposed memory device at 500 K have been confirmed to fall into the regime permissible for practical use.

## 1. Introduction

An integrated electronic system capable of operating at high temperature would be beneficial to various industrial applications, harsh environment systems, and core functional components for the aerospace systems [1,2]. When a semiconductor device is operated in a high-temperature environment, a number of problems mainly caused by the leakage currents due to greatly increased generation rate of electron-hole pairs (EHPs) are more likely to take place [3]. In order to resolve the issues, wide-bandgap materials such as GaN have been usually employed as the platform for the applications, instead of Si [4,5], by which the number of thermally generated carriers threatening the ideal electronic device performances can be reduced. Although there have been studies on high-temperature-operation transistors based on wide-bandgap materials towards the purpose, relatively less interest has been devoted to high-temperature memory technology. Additionally, although there is some research on nonvolatile memories [6,7,8], high-speed volatile memories coping with the processing unit in the specifically designed system have great deal of room to delve into. 

In this work, we develop a novel volatile memory having high-temperature operation capabilities. In most of the conventional one-transistor dynamic random-access memory (1T DRAM), the holes stored in the channel region modulate the threshold voltage and the drain current level in performing the read operation [9]. Additionally, in the conventional 1T DRAM device, the hole storage is provided by the energy barriers constructed by PN junctions at both ends of the channel and by the buried oxide (BOX). The stored holes can have several leakage paths, such as recombination, drift, diffusion, and inter-band tunneling [3]. 1T DRAMs in various novel structures have been proposed to suppress the data leakage and increase the retention time [10,11,12]. However, the previous studies are limited to room-temperature operation in most cases. An existing study introduces a 1T DRAM operating at high temperature, but the confirmed upper limit is 370 K and the subsequent studies are not active yet [13]. In this work, we propose, design, and characterize a novel 1T DRAM featuring a physical barrier made of wide-bandgap semiconductor material which confines the stored carriers highly effectively, verifying the memory operations through series of technology computer-aided design (TCAD) simulation works. 

## 2. Device Structure and Simulation Strategy 

**Figure 1:**
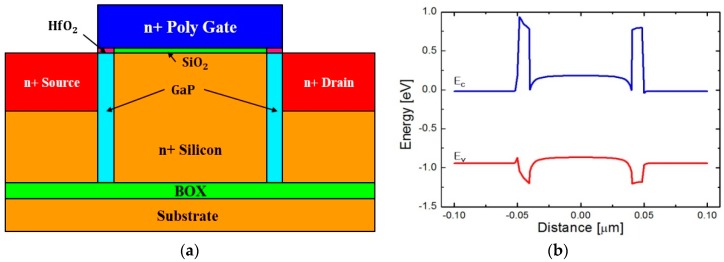
Device structure. (**a**) Schematic of the one-transistor dynamic random-access memory (1T DRAM) with partially introduced wide-bandgap barriers; (**b**) energy-band diagram along the channel direction in the proposed device.

The proposed 1T DRAM device with a pair of partially introduced vertical thin wide-bandgap barriers is illustrated in Figure 1a. As mentioned briefly, as temperature increases, junction leakage increases, and the stored holes smear out of the storage in most 1T DRAM devices [14]. Although the energy barrier formed by the gate oxide is as high as 3 eV, that introduced by a Si PN junction is at most the bandgap energy of Si, approximately 1.12 eV. Therefore, the leakage through source/drain junction becomes more prominent as temperature gets higher, owing to the carriers occupying the tail states of the Fermi-Dirac distribution with higher flatness about the Fermi level due to the temperature effect. In order to reduce the leakage currents stemming from the carriers coming over the energy barrier by PN junction, the 1T DRAM proposed in this work employs wide-bandgap material on both borders between channel and source/drain junctions. GaP has the least lattice mismatching among the single-species and compound semiconductors that can be used for device fabrication. We have sought the wide-bandgap materials with the highest degree of lattice matching for this application [15]. Unfortunately, GaP processing is not allowed in most of the Si CMOS clean rooms yet but molecular beam epitaxy (MBE) or metal-organic chemical vapor deposition (MOCVD) needs to be schemed for the epitaxial growth of GaP on Si. Si and GaP have similar thermal expansion coefficients of 2.6 × 10^−6^ K^−1^ and 4.65 × 10^−6^ K^−1^, respectively, at 300 K. Moreover, the thermal expansion coefficient of Si monotonically increases with temperature and that of GaP increases but shows a relatively slow slope, which makes both of them practically the same at the processing temperature above 800 K. Thus, the thermal expansion coefficient matching acts as another merit in forming the Si/GaP heterostructure. A more tangible effect of lattice matching and thermal expansion coefficient matching can be quantified as the interface trap density eventually. The interface trap densities between Si and GaP and between GaP and SiO_2_ were reported to be 1 × 10^13^ cm^−2^ and 7 × 10^12^ cm^−2^, respectively [16,17], which are comparably low as the trap density between Si and SiGe under a well-controlled epitaxial growth. This favorable interface status between Si and GaP results in the permissibly low off-state current in the metal-oxide-semiconductor field-effect transistor (MOSFET) operation. The wide-bandgap of GaP provides the energy barriers at both ends of the Si channel, which more effectively confines the carriers at high temperature compared with the energy barriers electrically formed by counter-doped Si regions [18]. SiC can be also adopted for our application in the sense that SiC has a stronger Si processing compatibility and wider energy bandgap than GaP. There are several different phases of SiC, but all the bandgap energies are larger than that of GaP, 2.26 eV. Thus, more effective carrier confinements become presumable with SiC for its application to high-temperature 1T DRAM technology. On the other hand, the lattice mismatch between Si and SiC is larger than that between Si and GaP regardless of the phases of SiC. Accurate control of the SiC barrier thickness should be performed in consideration of its epitaxial critical thickness on Si for being more affirmative with its application.

Figure 1b shows the energy-band diagram along the channel direction beneath the gate oxide. As can be confirmed by Figure 1b, since the large difference between bandgap energies of Si and GaP is mostly projected to the conduction band offset (CBO), the energy barrier seen by the conduction electrons is considerably higher than the barrier in the valence band, valence band offset (VBO), seen by the conduction holes. This high energy barrier in the conduction band effectively prevents the stored electrons from escaping to either source or drain junction, even at an elevated temperature. In order to make full use of the beneficial energy-band structure, the proposed 1T DRAM device stores electrons in a way different from that employed by most of the previously reported 1T DRAM, having a n^+^ channel to minimize unwanted loss of stored electrons by recombination in preserving the stored data. The electrical characteristics and the memory operations of the proposed 1T DRAM cell have been investigated by a commercial TCAD package, Sentaurus by Synopsys (Mountain View, CA, USA). Gate length is 100 nm, thicknesses of gate oxide and HfO_2_ are both 3 nm, thickness of BOX is 10 nm, and barrier width and depth are designed to be 10 nm and 75 nm, respectively. The doping concentrations of source/drain junctions and substrate are n-type 1 × 10^20^ cm^−3^ and n-type 1 × 10^16^ cm^−3^, respectively. A number of physical models including the Shockley-Read-Hall recombination model, Fermi statistics model, band-to-band tunneling model, and doping- and electric field-dependent mobility models are activated simultaneously in cooperation for higher accuracy and reliability of the simulation results. In particular, temperature models have been employed for reflecting the temperature effects. The highest operating temperature of a commercial memory device is known to be about 400 K. Considering the temperature robustness of metals used in the back-end-of-the-line (BEOL), the characterization and evaluation have been carried out at 500 K, which far extends the known upper limit of temperature warranting the permissible memory operations. 

## 3. Results

In typical MOSFET transistors and barrier-assisted 1T DRAMs, the energy barriers formed between the channel and the source/drain junctions control the off-state current. The role is taken over by the Si/GaP heterojunction in the proposed 1T DRAM device. Although the barrier introduced by the GaP heterojunction is even larger than that by the Si anisotype homojunction, the energy barrier can be effectively lowered by a high enough gate voltage. The tiny segments of HfO_2_ at both ends of the gate oxide are positioned to enhance the gate controllability over the barrier height of GaP, which is required for program operation which draws electrons from outside into the channel. 

**Figure 2:**
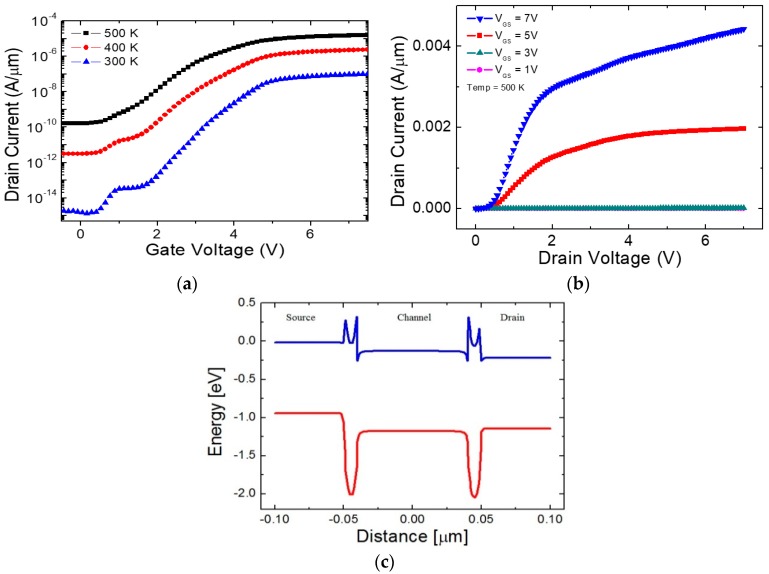
Operation characteristics. (**a**) I_D_-V_GS_ characteristic curves with V_DS_ = 0.2 V at 300 K, 400 K, and 500 K; (**b**) I_D_-V_DS_ characteristic curves at different V_GS_ values at 500 K; (**c**) energy-band diagram along the channel beneath the gate oxide at V_GS_ = 8 V and V_DS_ = 0.2 V at 500 K.

Figure 2a shows the I_D_-V_GS_ characteristic curves of the proposed 1T DRAM device at different temperatures of 300 K, 400 K, and 500 K. The current increases in both low and high V_GS_ regimes, which is owing to the increase of thermally generated carriers. Figure 2b demonstrates the output characteristic curves at different V_GS_ values. Operation voltage is higher than that of Si MOSFET with a comparable channel length since V_GS_ needs to be high enough to lower the high energy barrier brought by GaP for electron conduction. Focus in this work is made on the high-temperature operation capabilities of the proposed device eventually aiming the applications in the extremely harsh environment, and the dimension and drive voltage scaling criteria have not been taken forward. As can be confirmed by comparing Figure 1b and Figure 2c, applying a high enough V_GS_ lowers the energy barrier between the channel and the source/drain junctions. Additionally, since the electrons see an increase number of allowed energy states by the increased occupation probability determined by the Fermi-Dirac distribution at 500 K, a significant number of carriers overcome the energy barrier even though the higher energy barrier is constructed compared with the case of previously reported 1T DRAMs [3]. 

### 3.1. Program and Erase Operation Schemes

In order for the proposed device to operate as a 1T DRAM, it is necessary to create and store carriers to change the threshold voltage of the device. Electrons are created by impact ionization, which is one of the conventional carrier generation methods in 1T DRAM devices [19]. The proposed device differs from the existing ones in the sense that electrons are used instead of holes between carriers generated by impact ionization. As mentioned above, the energy barrier for the electrons constructed by Si/GaP heterostructure is larger than that for holes. In the erase operation, a large positive voltage is applied to the drain and a small negative voltage is applied to the gate. The detailed bias conditions for all the memory operations are summarized in Table 1. 

**Figure 3:**
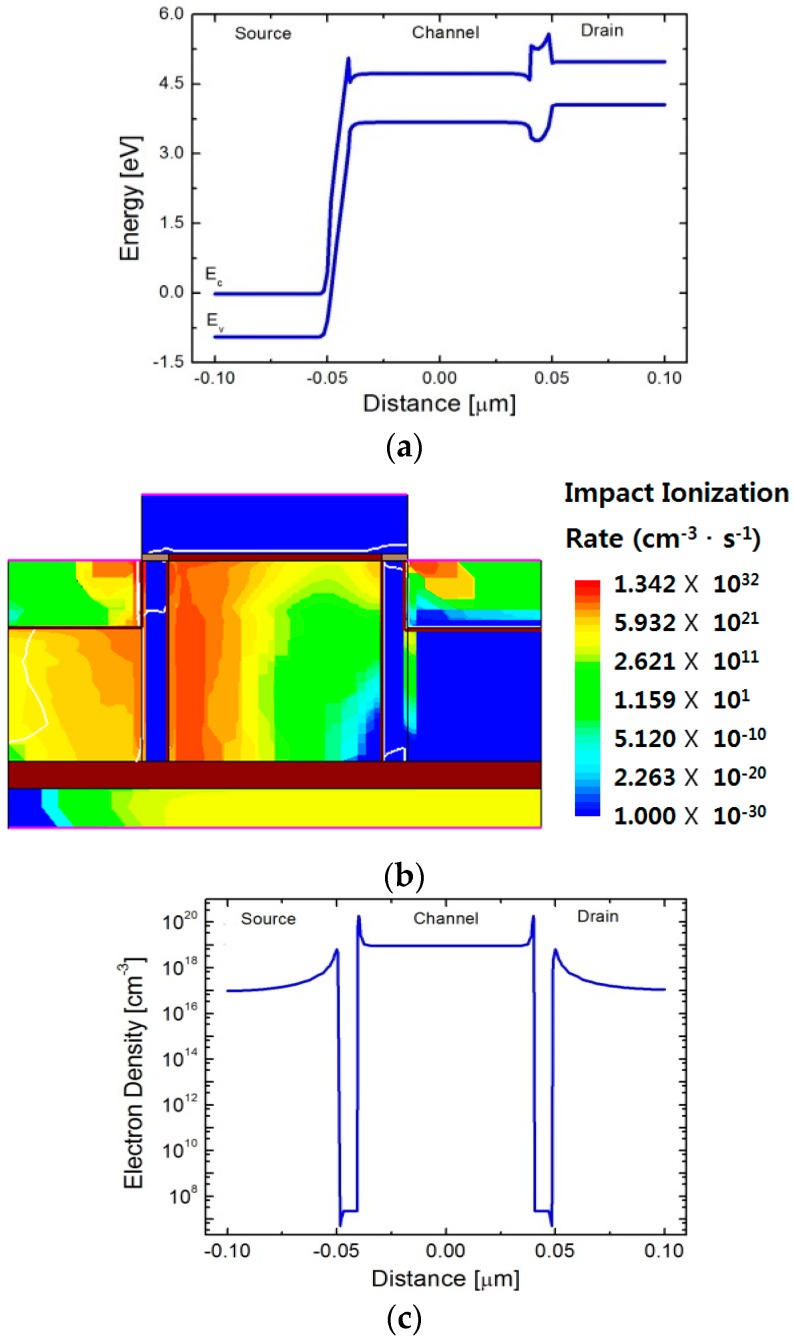
Verification of program operation. (**a**) Energy-band diagram along the channel direction under program bias condition at 500 K; (**b**) contour of impact ionization rate in the program operation at 500 K; (**c**) electron concentration after program operation at 500 K.

In order to give rise to impact ionization in the program operation, a large electric field should be applied in the channel direction. Figure 3a shows the simulated energy-band diagram under the high lateral electric field. The influences of the large-magnitude negative drain voltage and the small negative gate voltage are combined so that the energy barrier due to GaP substantially disappears. As the energy barrier introduced by GaP is lowered, it becomes possible to inject a large number of carriers into the channel while performing the program operation. 

The program operation can be confirmed by investigating the contour of the impact ionization rate over the device as demonstrated in Figure 3b. The high impact ionization rate is observed near the Si/GaP heterostructure near the source junction. The change in concentration of electrons stored in the channel after a program operation is depicted in Figure 3c. Electron concentration as high as 1 × 10^19^ cm^−3^ in the channel storage is assured even in the high-temperature environment at 500 K. 

**Figure 4:**
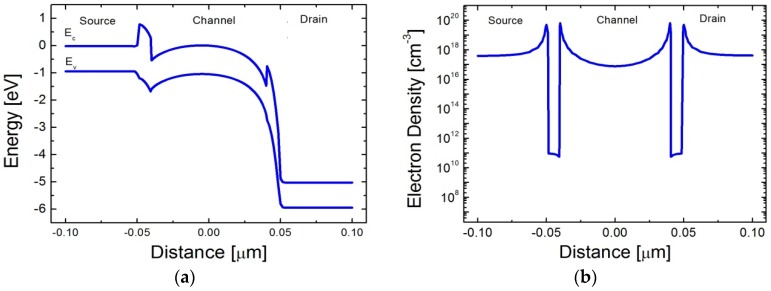
Verification of erase operation. (**a**) Energy-band diagram along the channel direction under the erase bias condition at 500 K; (**b**) Electron concentration after erase operation at 500 K.

For removing the stored electrons, a large positive voltage is applied to the drain junction. As shown in Figure 4a, since the energy barrier between the channel and the drain gets lower, the electrons in the channel are repelled to the drain junction. Additionally, a small negative gate voltage assists the repelling force on the electrons stored in the channel. As a result, the electron concentration in the channel is reduced as shown in Figure 4b, which can be clearly confirmed by comparison with Figure 3c. Since a massive amount of EHPs are generated at 500 K, even the lowest concentration of electrons in the channel region is higher than the channel doping concentration. The electron concentration increase by the EHPs degrades the read current ratio at the elevated temperature. 

### 3.2. Hold Operation and Retention Characteristics

In the 1T DRAM technology, hold operation can be optionally prepared to maintain the number of stored carriers, after program and erase operations before the read operation. For reducing the total power consumption over a period of memory operations, zero voltages are applied to both gate and drain terminals for holding the carriers stored in the channel.

**Figure 5:**
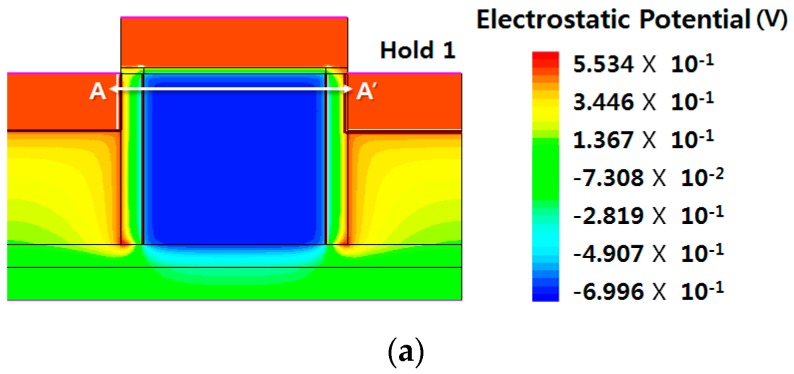

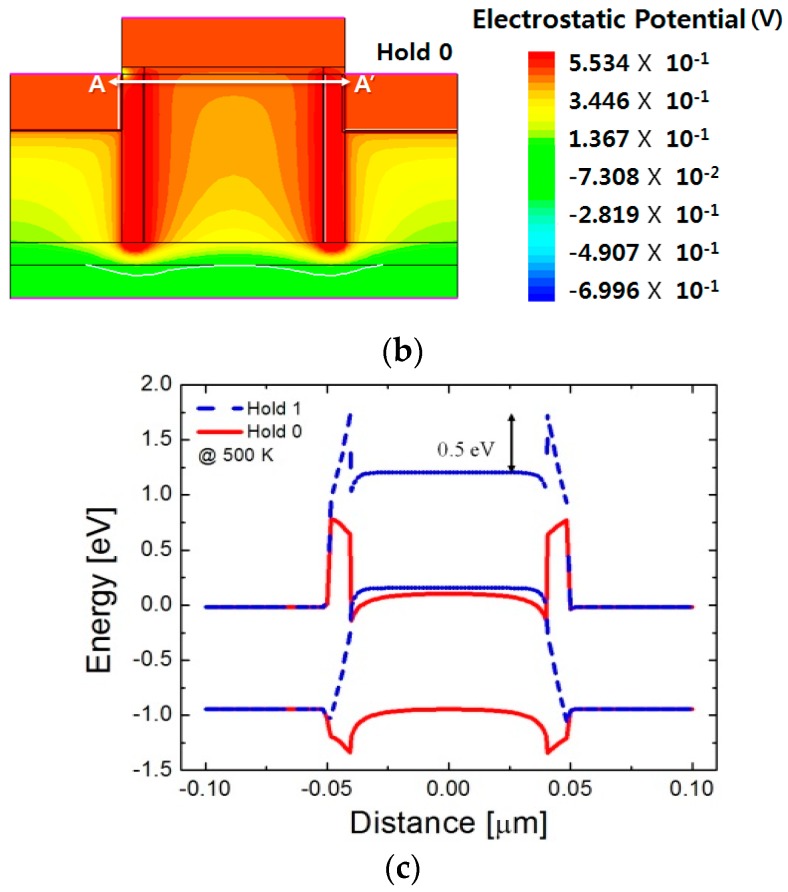
Verification of hold operation. (**a**) Potential distribution under the hold-1 condition; (**b**) potential distribution under the hold-0 condition; (**c**) energy-band diagrams under the hold-1 and the hold-0 conditions along the A-A’ cutline in (**a**).

Figure 5a,b shows the potential distribution and the energy-band diagram under the hold-1 and hold-0 conditions, respectively. In state 1, electrons are generated and the potential in the channel region is lowered. On the other hand, in state 0, the electrons are removed, and the potential is recovered to a high value leveling with the potentials in the source and drain junctions. Figure 5c depicts the energy-band diagrams in the channel direction in state 1 and 0 for the explicit comparison. The most important feature in the high-temperature operations is determined by whether a sufficiently high energy barrier can be steered or not so that the stored electrons are not allowed to escape to either source or drain junctions in state 1 at a high temperature. The original high electron energy in Figure 1b gets lower as electrons are accumulated in the channel as the program operation is progressed. However, as can be seen in Figure 5c, a high electron potential energy barrier of 0.5 eV still exists between the channel and the source/drain junctions in state 1. The energy barrier can be changed by introducing different wide-bandgap semiconductor material in the physical barrier with different energy bandgap and electron affinity. 

### 3.3. Transient Simulation Results for the Cyclic 1T DRAM Operations

**Figure 6:**
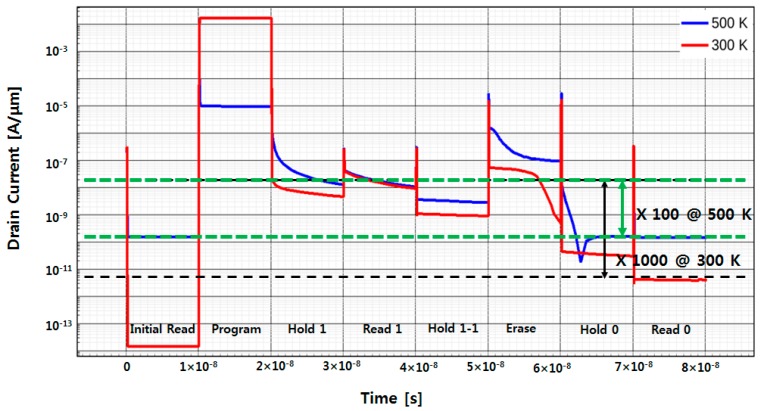
Transient simulation results for memory operations of the proposed 1T DRAM at 300 K and 500 K.

Figure 6 demonstrates the transient simulation results for 1-cycle memory operations of the proposed 1T DRAM. The cyclic operation consists of program/hold 1/read 1/hold 1/erase/hold 0/read 0/(hold 0) and the drain currents in each operation have been extracted at 300 K and 500 K. Read-1/read-0 current ratios are 1000 and 100 at 300 K and 500 K, respectively. Although the current ratio is reduced at 500 K compared with that at room temperature, not a small ratio is still preserved, and the ratio can be practically amplified and sensed by the supplemental functions of peripheral circuits. 

**Figure 7:**
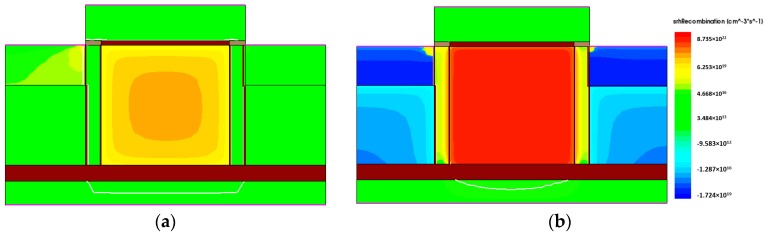
Distributions of the Shockley-Read-Hall (SHR) recombination rates under hold-1 condition at (**a**) 300 K and (**b**) 500 K.

The reasons that the current ratio decreases at 500 K can be considered to be the following: (1) Carrier recombination rate increases as the operating temperature increases as shown in Figure 7. For maintaining the steady-state carrier concentration, the recombination rate should be equal to the generation rate and the latter is a strong function of temperature. This also leads to reduction of carrier storage time in the end; (2) high temperature increases EHP generation, which results in higher electron concentration. Thus, the initial current level at 500 K should be significantly higher than that at 300 K as can be confirmed by Figure 6 along with the previous work [15]. Contemplating the quantitative analysis in Figure 6, the reason (2) is considered to be dominant. The carrier storage time, i.e., retention time is defined as the time moment when the read-1 and read-0 current ratio reduces down to 10%. Unlike the conventional DRAM where the retention is evaluated by the bitline voltage drop with time, state current reading should be performed for 1T DRAM where the bitline precharge scheme for read operation is not employed. By this method, the storage time is extracted to be 1 μs. At this moment, we have no idea how long or short this retention might be, since there is no concrete standard and requirement for 500 K operation at all yet. However, it is sure that the obtained retention time is much shorter than that of conventional DRAM in the 1-transistor 1-capacitor (1T1C) configuration.

## 4. Conclusions

We have proposed, designed, and characterized a novel 1T DRAM operational at 500 K featuring double wide-bandgap barriers for elongated data storage capability. The wide-bandgap semiconductor material, GaP, is introduced in the Si platform in order to heighten the energy barrier seen by the electrons stored in the channel storage. The series of simulation results support that the stored electrons in the channel are effectively preserved, even at 500 K. The firstly proposed scheme utilizing Si and lattice-matching wide-bandgap material GaP, and the device structure design, have prepared the strong potential for memory technologies in the high-temperature environment which can be found in the applications for auto-vehicles, industrial turbine systems, and aerospace systems. 

## Figures and Tables

**Table 1:** Bias conditions for program, erase, and hold operations.

Program		Erase		Read		Hold	
V_GS_	V_DS_	V_GS_	V_DS_	V_GS_	V_DS_	V_GS_	V_DS_
−0.5 V	−5.0 V	−0.5 V	5.0 V	0.5 V	0.2 V	0 V	0 V
